# Efficacy of propofol for the prevention of emergence agitation after sevoflurane anaesthesia in children: A meta-analysis

**DOI:** 10.3389/fsurg.2022.1031010

**Published:** 2022-10-03

**Authors:** Yinggang Xiao, Xuening Jin, Yang Zhang, Tianfeng Huang, Luojing Zhou, Ju Gao

**Affiliations:** ^1^ Medical College of Yangzhou University, Yangzhou, China; ^2^ Department of Anesthesiology, Institute of Anesthesia, Emergency and Critical Care, Yangzhou University Affiliated Northern Jiangsu People's Hospital, Yangzhou, China

**Keywords:** propofol, emergence agitation, child, sevoflurane, general anesthesia

## Abstract

**Background:**

Emergence agitation (EA) is a common postoperative behavioral disorder, predominantly in pediatric patients, after sevoflurane general anesthesia. This study was aimed at assessing propofol's efficacy and clinical conditions established for preventing EA in children under sevoflurane anesthesia.

**Methods:**

Randomized controlled trials (RCTs) that comparatively investigated propofol and control treatment in terms of efficacy and safety on administration at the end of surgery and examinations to prevent EA in children under sevoflurane anesthesia were searched. The sources accessed included PubMed, Embase, Cochrane Central Register of Controlled Trials, and ClinicalTrials.gov. Furthermore, manual searches were performed to identify studies; the last review was conducted on March 21, 2022. When the risk of bias assessment of trials was performed with the Cochrane Risk of Bias Tool, we calculated risk ratios (RRs) with 95% confidence intervals (CIs) for EA incidence and mean differences (MDs) with 95% CI for continuous data.

**Results:**

We included 12 RCTs with 1103 children. EA incidence (RR: 0.51, 95% CI: 0.39 to 0.67) and Pediatric Anesthesia Emergence Delirium scores (MD: −3.14, 95% CI: −4.37 to −1.92) were lower in the propofol group. Subgroup analyses showed lower EA incidences with 3 mg/kg propofol (RR: 0.22, 95% CI: 0.13 to 0.38) without extension of the PACU time (MD: 4.97, 95% CI: −0.84 to 10.78) in the laryngeal mask airway (LMA; RR: 0.52, 95% CI: 0.36 to 0.77) and spontaneous breathing (RR: 0.36, 95% CI: 0.21 to 0.62) groups.

**Discussion:**

We confirmed that a prophylactic dose of propofol prevented EA and decreased its severity in children under sevoflurane anesthesia. Furthermore, several conditions such as 3 mg/kg propofol, LMA, and spontaneous breathing, potentially contributed to EA prevention.

**Systematic Review Registration:**

https://www.crd.york.ac.uk/prospero/display_record.php?RecordID=274692, identifier: PROSPERO (No. CRD42021274692).

## Introduction

Because of hemodynamic stability, no airway irritation, and rapid emergence, sevoflurane has become the most common inhaled volatile anaesthetic agent used in children (1). However, sevoflurane administration is strongly associated with emergence agitation (EA), the incidence of which in children under sevoflurane general anaesthesia varies between 10% and 80% (2). EA is defined as a postoperative behavioural disorder mainly occurring in children. It is characterized by inconsolability, incoherence, thrashing, restlessness, and non-purposeful movement (3). Emergence delirium (ED) is a severe subtype of EA and defined as a dissociative state of consciousness. Often, EA and ED are used interchangeably (4). Researchers have proposed various scoring systems to diagnose and quantify EA, including the Cravero scale (5), Aono's four-point scale (6), five-step EA scale (7), four-point scale (1), and Watcha scale (8). However, these scales have not been psychometrically tested, and they use emotional distress and psychomotor agitation as alternative markers of psychosis, which reduces their accuracy and limits their use (3). Most importantly, they do not include the most important confounding variable–pain that confuses those who are evaluating ED. Currently, paediatric anaesthesiologists generally consider the psychologically tested Pediatric Anesthesia Emergence Delirium (PAED) scale to be the most effective and reliable (Supplementary Table S1) (3, 9). The scale contains cognitive and arousal assessment items and is used in children over the age of 2 years. It is generally believed that its score is greater than or equal to 10 points, and its diagnostic sensitivity and specificity for postoperative agitation are the highest in the PICU (10).

Propofol is a widely used short-acting intravenous agent for sedation and induction and maintenance of anaesthesia. It can also be injected at the end of a procedure or examination to decrease the incidence and severity of EA (4, 6, 8). However, it remains unclear whether and how the propofol dose, the process of general anaesthesia, pain and the more detailed classification of surgery and examination play critical roles when propofol is used to prevent EA. These factors may intuitively affect postoperative agitation and have been reported in the literature. Considering these deficiencies in the literature and given that some random control trials (RCTs) have shown the preventive effects of propofol against EA under various conditions and processes, we conducted a meta-analysis of recent RCTs to achieve deeper insight into the problems listed above.

## Materials and methods

### Search strategy and selection criteria

The Preferred Reporting Items for Systematic Reviews and Meta-Analyses (PRISMA) statement guided our meta-analysis, the protocol of which was prospectively registered on the international prospective register of systematic reviews (no. CRD42021274692). Two authors independently included all the relevant studies by searching PubMed, Embase, Cochrane Central Register of Controlled Trials, and ClinicalTrials.gov from the inception of these sources until March 21, 2022. Furthermore, a manual search of relevant citations from meta-analyses associated with our objective was performed to include literature that had not been retrieved in the database search. There are no language restrictions in the retrieval process. The keywords used were “propofol’, “children”, “sevoflurane”, and “emergence agitation”. The PRISMA 2020 checklist and PRISMA 2020 for Abstracts Checklist for this meta-analysis has been included in Supplementary Data Sheets 1, 2. Supplementary Data Sheet 3 shows the full search strategies employed for the three databases and ClinicalTrials.gov.

### Inclusion and exclusion criteria

The following inclusion criteria were developed based on the Population, Intervention, Comparison, Outcome, and Study design (PICOS) framework. RCTs in children under sevoflurane general anaesthesia, in which propofol was injected intravenously at the end of surgery or examination to prevent EA, in which the control group received a placebo or no intervention, and in which EA incidence was reported were included. Studies in which only the efficacy of propofol combined with other drugs was investigated were excluded. Furthermore, duplicates and studies that were judged as lacking sufficient data after checking relevant information on the registration platform and e-mailing relevant authors were discarded. All articles were sorted and classified using Endnote X9 as the bibliography database manager.

### Data extraction

A data extraction form was designed and adjusted by pre-extraction. Thereafter, two investigators independently extracted three aspects of the included studies: basic information; research methods for quality assessment; and information for forest plot and subgroup analysis including premedication, dose and time of propofol injection, surgery or examination type, nerve block, ventilation mode, type of airway establishment, diagnostic criteria for EA, awakening time, time in the PACU, incidence of EA, and PAED scores and time of measurement. When authors only showed correlative data in graphs, relevant data were extracted using WebPlotDigitizer (https://automeris.io/WebPlotDigitizer/), a web-based tool for the data extraction from plots, images, and maps. After cross-checking, the investigators discussed discrepancies, and final decisions were made by another chief physician.

### Risk of bias assessment

Two independent reviewers performed the risk of bias assessment for RCTs by using the Cochrane Risk of Bias Tool. Discrepancies were resolved by discussion between the reviewers, and results were summarized using Review Manager (RevMan) 5.4.1.

### Data synthesis and statistical analysis

All data synthesis and statistical analyses were performed using Stata/MP 16.0. The primary outcome was EA incidence. Pooled RR with 95% CI was calculated to analyse the proportion of children with EA. PAED scores, awakening time, and time in the PACU as continuous data were the secondary outcomes. Pooled mean differences (MDs) with 95% CIs were calculated to compare the continuous data for these parameters between groups.

All included RCTs reported EA incidence, which resulted in data synthesis based on dichotomous variables in this study. PAED scores, awakening time, and time spent in the PACU that were reported in the form that was appropriate for this meta-analysis were synthesized as continuous variables. When PAED scores were not in the form of mean ± standard deviation (SD), they were converted accordingly. The other two outcomes in articles had appropriate form. Intragroup 95% CIs were converted to the corresponding SDs or calculated SDs with all individual PAED scores extracted from graphs. The number of participants with ED was not reported in some RCTs; this was obtained by calculations involving the incidence of ED and the total number of participants. For studies with multiple groups, only the data of the propofol and control groups were synthesized and analysed.

The random-effects model (DerSimonian-Laird method) was used for meta-analysis. Forest plots were used to visually display the results of individual articles and syntheses. Firstly, the L'abbé plot was used to check for heterogeneity with binary data. The Cochran Q test and *I*^2^ testing were also used to assess heterogeneity. *I*^2^ > 50% indicated moderate-to-high heterogeneity, and a Cochran Q test *P* < 0.1 was considered indicative of significant heterogeneity. To assess significant heterogeneity among the study results, subgroup analyses were performed on the basis of possible and clinically meaningful causes (dose of propofol, method of airway establishment, ventilation method, type of procedure and examination, pain, premedication, and caudal block). When non-painful MRI examinations were categorized into one group, surgery, which is painful, was categorized in another group. Grouping was performed on the basis of whether premedication affected cognition. A contour-enhanced funnel plot was used to assess publication bias, and a nonparametric trim-and-fill analysis was performed to estimate if the results of this meta-analysis would be disturbed by the bias. The robustness of the synthesized results was evaluated by performing the trim-and-fill analysis without drawing a diagram.

### Certainty assessment

The Grading of Recommendations Assessment, Development and Evaluation (GRADE) framework was used to assess certainty in the body of evidence for EA incidence and PAED score among the studies by using GRADEpro 3.6. The criteria for certainty were as follows: risk of bias, inconsistency, indirectness, imprecision, and publication bias. The evidence was divided into four grades: high quality, moderate quality, low quality, and very-low quality.

## Results

### Study selection and characteristics

**Figure 1:**
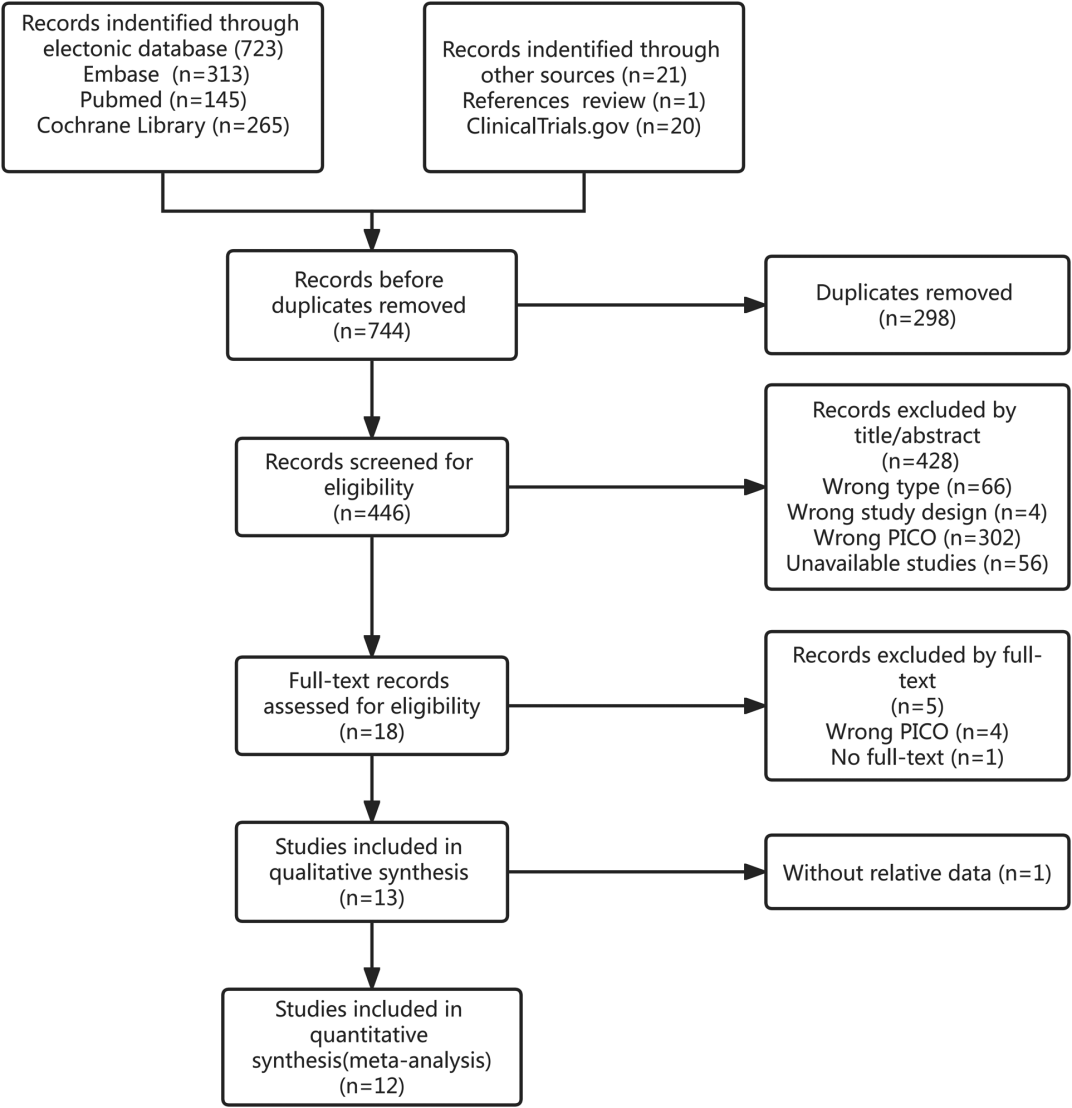
The flow diagram about the study retrieval process.

Through database and manual searches, we initially obtained 744 records. After the exclusion of duplicate studies and preliminary screening based on titles/abstracts and re-screening of full texts, 13 articles were included in the qualitative synthesis. One of the articles reported neither the incidence of EA nor PAED scores (11); thus, 12 articles were included in the final meta-analysis (1, 4, 6–8, 12–18). One article (12) which did not include “sevoflurane” in the title and abstract was obtained by a manual search based on a 2015 meta-analysis (19). The total data of 1103 children (551 and 552 in the propofol and control groups, respectively) aged 1 to 13 years with an American Society of Anesthesiologists physical status (ASA) of I or II were included in our analyses. Only seven studies involved the evaluation of PAED scores and provided related data (4, 6, 8, 12, 13, 17, 18). In two of these studies, PAED scores were extracted from graphs by using WebPlotDigitizer and then calculated (4, 12). One trial published in the Korean Journal of Anesthesiology was written in Korean but had an English title, abstract, and forms, which meant that relative information and data could be accessed using Google Translate where necessary (15). Figure 1 shows the flow diagram for study inclusion, and Table 1 shows the characteristics of the included studies.

**Table 1:** Basic characteristics of included studies.

Study	Age (years)	Type of surgery	Propofol/Control	Dose (mg/kg)	Premedication (mg/kg)	Airway	Ventilation	Caudal block
Aouad 2007 (19)	2.0–6.0	Strabismus surgery	41/36	1.0	Midazolam 0.5	LMA	Mechanical	No
Kim 2008 (16)	2.0–7.0	Strabismus surgery	30/30	1.0	No	LMA	Mechanical	No
Abu-Shahwan 2008 (15)	2.0–7.0	MRI	42/41	1.0	No	LMA	Mechanical	No
Lee 2010 (14)	3.0–8.0	Adenotonsillectomy	44/44	1.0	Thiopental sodium 1	Intubation	Mechanical	No
Kim 2011 (1)	1.0–13.0	Strabismus surgery	31/35	1.0	Atropine 0.01	Intubation	Mechanical	No
Kim 2013 (6)	1.7–6.0	Inguinal hernia repair	69/70	1.0	No	LMA	Spontaneous	No
Ali 2013 (18)	2.0–6.0	Adenotonsillectomy	40/40	1.0	Midazolam 0.5	Intubation	Mechanical	Yes
Rashad 2014 (7)	1.0–3.0	Ambulatory hypospadias repair	20/20	1.0	No	LMA	Spontaneous	No
Costi 2015 (4)	1.0–12.0	MRI	109/109	3.0	Midazolam 0.5	LMA	Spontaneous	Yes
Bong 2015 (13)	2.0–7.0	MRI	39/41	1.0	No	LMA	Spontaneous	Yes
Abbas 2019 (8)	1.0–12.0	Inguinal hernia repair	32/32	3.0	No	LMA	Spontaneous	No
Ramlan 2020 (17)	1.0–5.0	Lower abdominal, Craniomaxillofacial, Orthopedic surgery and Dental	54/54	0.5	Ketamine 0.5	LMA/intubation	Mechanical	No

Five studies appeared to meet the inclusion criteria but were excluded (20–24). One study did not have a control group (22), and investigators used propofol after the induction of anaesthesia in another study (20). In two studies, propofol was injected with fentanyl and tramadol in the propofol groups, respectively (21, 23). As native speakers of Chinese, we evaluated the findings from one of the studies, which was written in Chinese (21). The final study was excluded because the full text could not be accessed in any way (24). Supplementary Figure S1 presents assessments of the risk of bias for each included study.

### Meta-analysis

#### Analyses based on the pooled effect size

**Figure 2:**
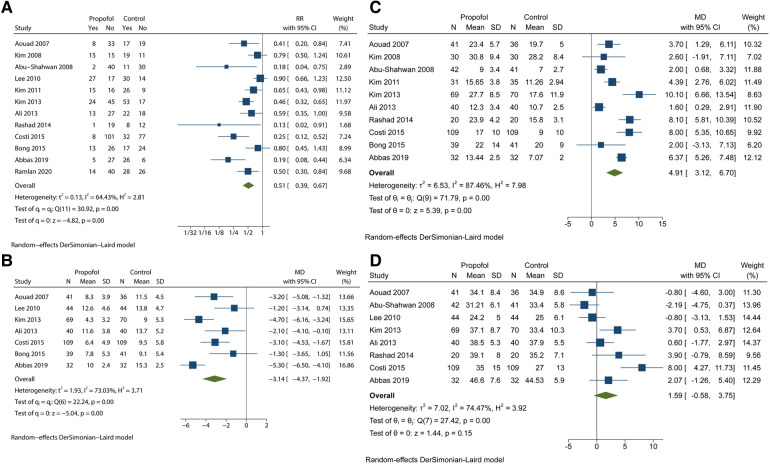
Analyses based on the pooled effect size. (**A**) the meta-analysis for the EA incidence. (**B**) the meta-analysis for the PAED scores. (**C**) the meta-analysis for the awakening time. (**D**) the meta-analysis for the PACU time.

We initially analysed all 12 studies based on the incidence of EA by using the forest plot. The quality of the studies was high. Supplementary Table S2 includes details of the evidence certainty based on GRADE. A statistically significant reduction was observed in the incidence of EA in the propofol group in comparison with that in the control group (RR = 0.51; 95% CI = 0.39 to 0.67; *P* < 0.05), with moderate heterogeneity (*I*^2 ^= 64.43%; *P* < 0.1) (Figure 2A). Moreover, the variation in PAED scores is summarized in Figure 2B, which revealed that propofol administration at the end of procedure or examination attenuated the severity of EA in children under sevoflurane anaesthesia (MD = −3.14; 95% CI = −4.37 to −1.92; *P* < 0.05). On the basis of the results of the *I*^2^ test and Q test (*I*^2 ^= 73.03%; *P* < 0.1), we confirmed high heterogeneity among the seven trials. The moderate quality of PAED scores was assessed by GRADE (Supplementary Table S3). Substantial heterogeneity weakened the confidence in the estimate of effect. Statistical difference was observed in the awakening time of the propofol group in comparison with that in the control group (MD = 4.91; 95% CI = 3.12 to 6.70; *P* < 0.05), with high heterogeneity (*I*^2 ^= 87.46%; *P* < 0.1) (Figure 2C). In addition, there was no significant difference in PACU time whose CI included invalid line between the two groups (MD = 1.59; 95% CI = −0.58 to 3.75; *P* > 0.1), with high heterogeneity (*I*^2 ^= 87.46%; *P* < 0.1) (Figure 2D). The L'abbé plot showed that most of the circles representing studies were distributed along the estimated line (Supplementary Figure S2).

#### Subgroup meta-analyses

**Figure 3:**
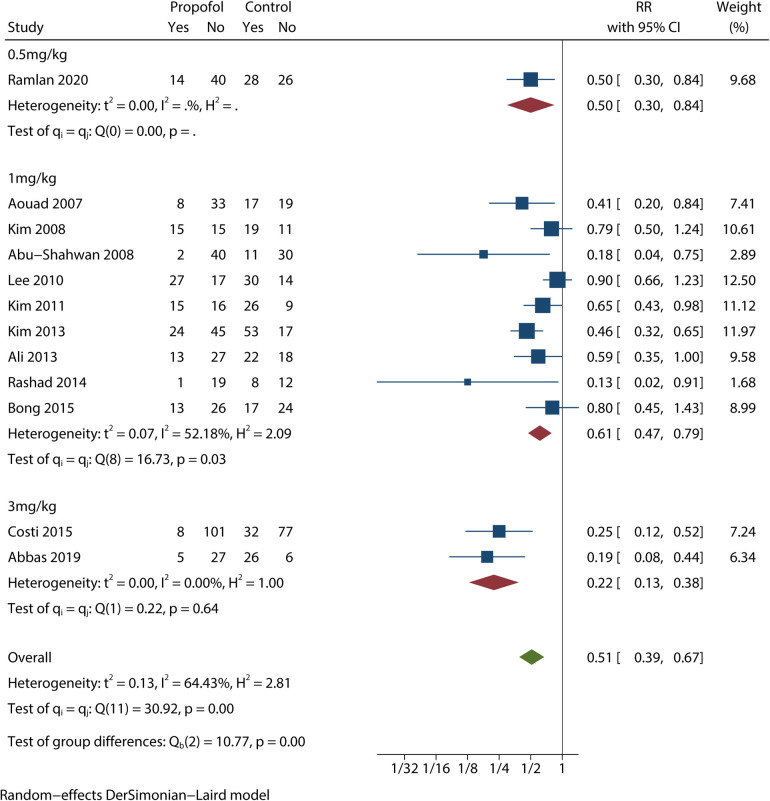
Subgroup analysis for EA incidence based on the dose of propofol.

Since the test of group differences showed *P* < 0.1, a subgroup analysis based on the dose of propofol showed statistically significant differences between the 1 mg/kg and 3 mg/kg groups, with a marked decrease in heterogeneity (Figure 3). While the 1 mg/kg propofol group showed similar results (RR = 0.61; 95% CI = 0.47 to 0.79) with moderate heterogeneity (*I*^2 ^= 52.18%; *P* < 0.1), the 3 mg/kg propofol group showed results (RR = 0.22; 95% CI = 0.13 to 0.38) with no heterogeneity (*I*^2 ^= 0%; *P* > 0.1). Another 0.5 mg/kg group that only included one trial (16) showed RR = 0.50 with 95% CI = 0.30 to 0.84. Most importantly, the 95% CIs of the 1 mg/kg and 3 mg/kg groups did not overlap each other.

**Figure 4:**
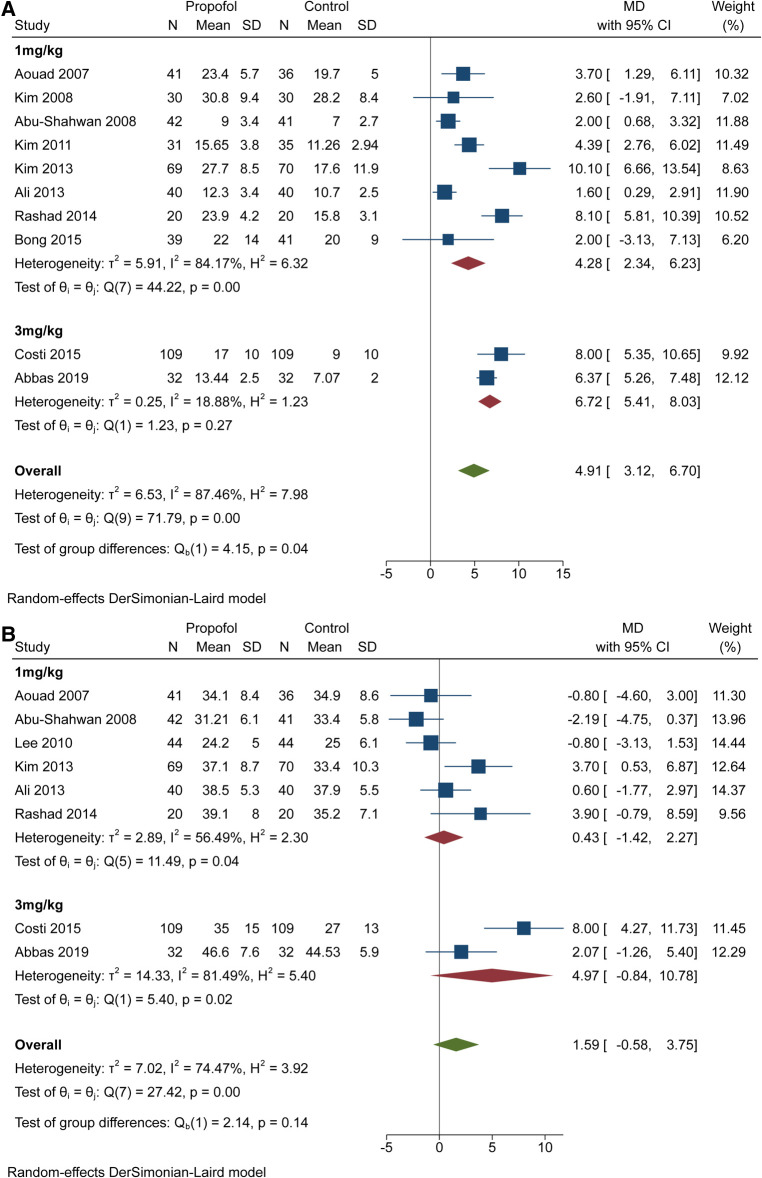
Subgroup analysis for recovery time based on the dose of propofol. (**A**) subgroup forest plot for awakening time. (**B**) subgroup forest plot for PACU time.

Since the propofol dose may influence the recovery time, we performed two subgroup analyses. For awakening time, results with significant differences (*P* < 0.1) were obtained (Figure 4A). The 1 mg/kg group showed MD = 4.28 and 95% CI = 2.34 to 6.23 with high heterogeneity (*I*^2 ^= 84.17%, *P* < 0.1). The 3 mg/kg group showed MD = 6.72 and 95% CI = 5.41 to 8.03 with low heterogeneity (*I*^2 ^= 18.88%, *P* > 0.1). For time in PACU, we obtained results without statistical differences (*P* > 0.1) and both subgroups showed 95% CIs that overlapped invalid line like before (Figure 4B). Furthermore, the 95% CI (−0.84 to 10.78) of the 3 mg/kg group covered the value of MD (0.43 95% CI = −1.42 to 2.27) in 1 mg/kg group.

**Figure 5:**
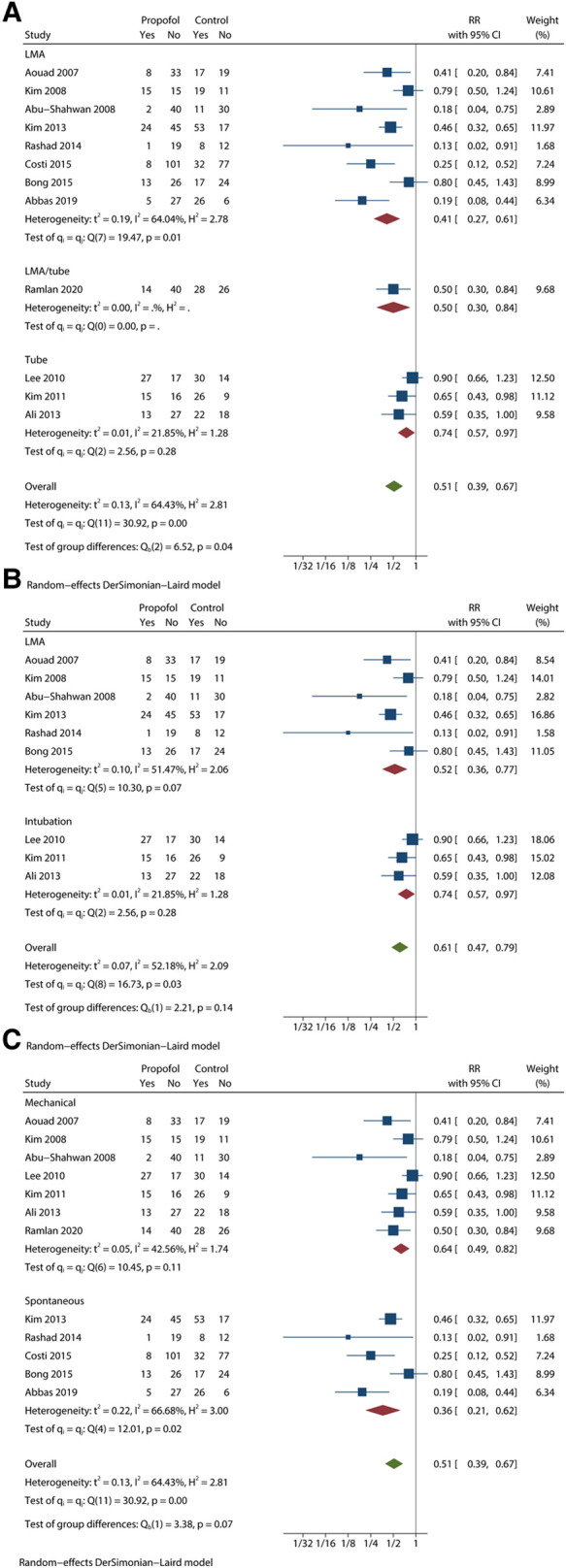
Subgroup analysis of EA incidence based on other conditions with meaningful results. (**A**) subgroup forest plot based on airway establishment. (**B**) subgroup forest plot based on airway establishment among studies that used 1 mg/kg propfol. (**C**) subgroup forest plot based on ventilation mode.

We also found a meaningful result in the subgroup analysis based on airway establishment technique (Figure 5A). In the test of group differences (*P* < 0.1), the participant's airway was maintained by LMA insertion or endotracheal intubation. Except Ramlan et al. (16), who used both techniques, the included trials only performed one of the two techniques. The LMA/intubation group showed RR = 0.50 with 95% CI = 0.30 to 0.84. The LMA group showed moderate heterogeneity (*I*^2 ^= 64.04%; *P* < 0.1) results (RR = 0.41; 95% CI = 0.27 to 0.61), but the intubation group showed more homogeneous results (RR = 0.74; 95% CI = 0.57 to 0.97) with low heterogeneity (*I*^2 ^= 21.85%; *P* > 0.1).

Considering the moderate heterogeneity in the LMA group, we synthesized and analysed studies that used 1 mg/kg propofol (Figure 5B). In the LMA group, *I*^2 ^= 51.47%, *P* < 0.1, RR = 0.52 and 95% CI = 0.36 to 0.77. The intubation group showed perfectly consistent results. Although the heterogeneity was successfully reduced, the difference between LMA and intubation almost disappeared (*P* > 0.1).

For ventilation mode, we obtained results with significant differences (*P* < 0.1) (Figure 5C). The mechanical group showed RR = 0.64 and 95% CI = 0.49 to 0.82 with low heterogeneity (*I*^2 ^= 42.56%, *P* > 0.1). The spontaneous group showed RR = 0.36 and 95% CI = 0.21 to 0.62 with low heterogeneity (*I*^2 ^= 66.68%, *P* < 0.1).

The results of other subgroup analyses did not appear to be significant, with a huge overlap among 95% CIs and *P* > 0.1 in the test of group differences (Supplementary Figures S3–S6). Heterogeneities were not downgraded by subgroup analyses based on the type of surgery and examination, premedication, caudal block, and pain.

### Publication bias

Supplementary Figure S7 illustrates the contour-enhanced funnel plot with trim-and-fill analysis based on the incidence of EA. The existence of publication bias was evidenced by the fact that most of the circles representing included studies were concentrated on the estimated line but were asymmetrical to the line. Moreover, the two imputed studies existed in the grey area in the centre, which indicated that two trials with negative results might not have been published. However, by performing trim-and-fill analysis without drawing a diagram, we demonstrated that the results were robust. Even with the addition of imputed studies, the pooled effect size suggested an advantage in the propofol group (RR = 0.54; 95% CI = 0.41 to 0.71).

## Discussion

This meta-analysis investigated the EA-preventing effects of propofol administration at the end of procedures or examinations under sevoflurane general anaesthesia in children. Considering the incidence of EA, our pooled effect size (RR = 0.51) of 12 studies was situated at the left of the invalid line, and the 95% CI of 0.39 to 0.67 did not overlap zero. This result suggests that propofol appears to prevent EA in children who receive sevoflurane for general anaesthesia. Developed in 2004, the PAED scale is used most commonly to evaluate EA/ED in children aged >2 years (9). In the forest plot of seven studies recording PAED scores, the diamond representing MD (−3.14) and 95% CI (−4.37 to −1.92) was located at the left of the invalid line, which indicates that the PAED scores in the propofol group are much lower than those in the control group. Thus, propofol can attenuate the severity of EA. Some studies hold the view that the incidence of EA after sevoflurane anaesthesia is higher than that after propofol or other inhalational anaesthesia (5, 25, 26). Therefore, we focused on EA that occurred in sevoflurane anaesthesia.

Recent studies have shown consistent improvements in our understanding of the pathogenesis of sevoflurane-induced EA. Sevoflurane significantly affects *γ*-aminobutyric acid (GABA) metabolism in the central nervous system (CNS) and directly inhibits the CNS (27). After discontinuation of sevoflurane, rapid synaptic metabolism of GABA causes short-term normalizations of GABA levels, resulting in vanishing inhibition of the CNS and focal central sensitization. These factors cause decremental responses to sensory stimuli, a state of euphoria in the CNS, and eventual EA. The effects of sevoflurane on the locus coeruleus (28) and ion channels (29) are correlated with EA. Additionally, Jacob (30) found that lactate and glucose concentrations in the brain increased after sevoflurane anaesthesia, which results in EA. When GABA was metabolized quickly at the end of sevoflurane anaesthesia, investigators administered propofol as a GABA receptor agonist to delay awakening and sedate patients to inhibit excitatory synapses to prevent EA (16, 31).

Considering the significant heterogeneity (*I*^2 ^= 64.43%, *P* < 0.1) that was also suggested by the L'abbé plot, we performed a series of subgroup analyses based on factors intuitively affecting EA. First, trials were clustered by the dose of propofol. Although we were unable to determine heterogeneity in the 0.5 mg/kg group, which only included one study (16), the heterogeneities of the two other groups became lower (*I*^2 ^= 52.18% and 0%). Furthermore, the 3 mg/kg group with no heterogeneities (*P* > 0.1) showed a lower RR value than the 1 mg/kg group, and 95% CIs of the two groups did not overlap each other. These findings indicate that injections of 3 mg/kg propofol have a better effect on EA reduction. The results of the test of group differences (*P* < 0.01) strengthened this idea. In addition, investigators in the 3 mg/kg group did not administer a single injection of 3 mg/kg propofol but instead administered a propofol 1-mg/kg IV bolus to the children with another 2 mg/kg given over the following 3 min (4, 8).

Quite a few studies have demonstrated that prolonging recovery time can reduce the incidence of EA after sevoflurane (32, 33). The advantage of propofol over dexmedetomidine and midazolam is the minor influence of awakening time or discharge time (34–36). Therefore, the balance between efficacy and rapid recovery or discharge from PACU should be considered while administering higher doses. First, we synthesized and analysed the awakening time and time spent in the PACU. The diamond representing the MD (4.91) and 95% CI (3.12 to 6.70) was located at the right of the invalid line, suggesting that propofol extended the wakening time. However, it did not extend the time spent in the PACU, indicating the clinical effectiveness of propofol for the reduction of EA. Next, it was important to ensure whether higher doses of propofol influenced the recovery time, which led to two subgroup analyses. Figure 4A showed that CIs in the two subgroups overlapped obviously, but the corresponding values for group differences indicated statistical significance (*P* < 0.5). Meanwhile, the value of heterogeneity decreased from 87.46% to 84.17% and 18.88%, suggesting that differences in the dose of propofol would influence awakening time. Although the extension of time weakened the clinical applicability of 3 mg/kg propofol, another subgroup analysis yielded encouraging findings. Both diamonds representing the pooled effect size with the 95% CI in the two subgroups overlapped the invalid line, indicating that 1 mg/kg and 3 mg/kg propofol would not prolong the time in the PACU. Most importantly, the diamond (MD = 4.97; 95% CI = −0.84 to 10.78) of the 3 mg/kg group included the MD (0.43) of the 1 mg/kg group, and the related test of group differences (*P* > 0.1) indicated no discrepancy in the PACU time between different doses of propofol. Thus, 3 mg/kg propofol was better than 1 mg/kg propofol for the prevention of EA.

Another subgroup analysis showed the difference between LMA and endotracheal intubation (*P* < 0.05). In comparison with the intubation group (RR = 0.74; 95% CI = 0.57 to 0.97), the LMA group (RR = 0.41; 95% CI = 0.27 to 0.61) with lower heterogeneity (*I*^2 ^= 21.85%; *P* > 0.1) showed a better preventive effect of EA. Only one study in which 0.5 mg/kg propofol was injected used two approaches for the airway establishment, which made it impossible to calculate heterogeneity and compare the findings (16). However, there was a slight decrease in the heterogeneity of the LMA group (*I*^2 ^= 64.04%; *P* < 0.05). Since the dose of propofol potentially influences the incidence of EA, we decided to perform another subgroup analysis without studies using 3 mg/kg propofol. In this new assessment, the *I*^2^ of the LMA group dropped 13 percentage points, from 64.04% to 51.47%. Thus, the method used for airway establishment may be one of the causes of heterogeneity. This result confirms our previous findings and suggests that propofol in paediatric patients with ventilation *via* LMA was more effective for preventing EA. As a form of non-invasive ventilation, LMA results in greater hemodynamic stability with less irritation and adverse events (37, 38). In comparison with intratracheal intubation, LMA is expected to reduce agitation scores. Meanwhile, propofol may be more effective in calm patients with less irritation.

The last subgroup analysis with positive results (*P* < 0.1) was based on the distinction between spontaneous breathing and mechanical ventilation. Propofol in the spontaneous ventilation group (RR = 0.36; 95% CI = 0.21 to 0.62) seems to be more effective than in the mechanical ventilation group (RR = 0.64; 95% CI = 0.49 to 0.82). However, these factors seem less reliable than others. The mechanical ventilation group showed a higher heterogeneity (*I*^2 ^= 66.68%, *P* < 0.1) than the pooled *I*^2^ of 64.43%, which indicates that ventilation was not the source of heterogeneity. Moreover, the 95% CI of the spontaneous ventilation group seemed to cover the RR of the mechanical ventilation group.

Premedication (*P* = 0.84), caudal block (*P* = 0.98), type of procedure or examination (*P* = 0.17), and painful surgery (*P* = 0.45) did not influence the preventive effect of propofol. They were not causes of heterogeneity since no decrease in heterogeneity was noted in the related subgroup analyses. The result suggesting that EA was unaffected by these factors is well supported by some studies (39–44). More than 14% of children showed no response to oral midazolam and obvious restlessness instead (43). Until now, the evidence for proving that benzodiazepines can decrease the incidence and severity of EA/ED has been lacking (41, 42). One review supported that the type of procedure or examination as a preoperative factor could not influence the incidence of ED (40). Regional blocks can attenuate postoperative pain and reduce anaesthetic requirements, but there is inadequate data to suggest that they are associated with reduction of EA in patients under sevoflurane anaesthesia (39, 44). From raising questions, defining inclusion and exclusion criteria, searching literature, data analysis, to the final full text writing, we have fully complied with the most standard requirements.

This meta-analysis had some limitations. Although subgroup analyses decreased the value of *I*^2^, low to moderate heterogeneity still existed across most subgroups, which weakened the reliability of the results. First, the diagnostic criteria for EA were different among the included trials. The most common scale was PAED. While Abu-Shahwan (14) suggested an EA cut-off score of 16 or greater on the scale, three studies (12, 16, 18) diagnosed EA for scores ≥ 10 and Costi (4) adopted a score of 12 as the cut-off. Moreover, Aono's scale (6, 17), five-step EA scale (6, 7), 4-point scale (1), and Watcha scale (4, 8) were used. Some trials used multiple scales along with the PAED scale as the criteria of EA severity. In addition, the PAED scale had some limitations, including subjective assessment and overlap with pain presentation. Second, the trials were conducted with different patient age ranges and processes of general anaesthesia, including parental company, analgesic drugs, and timing of LMA removal or extubation. Third, the participants came from different countries and continents. Finally, limited by original literature, we were unable to extract baseline and pain scores to address concerns about false positives, but all included studies declared that they only recruited subjects without mental and neurologic disease. We believe that discrepancies in diagnostic criteria are the most important factor causing significant heterogeneity and recommend the use of the PAED scale for unified criteria in EA/ED research. Although we regarded RR and MD as the pooled effect size to address discrepancies in baseline data that were caused by the diagnostic criteria for EA, anaesthesia procedures, age, and other reasons, it is undeniable that they had some effect on the credibility of the conclusion, as shown in the heterogeneity test. Moreover, we indicate that 3 mg/kg propofol is more effective to prevent EA, but more studies with 3 mg/kg propofol are needed.

In conclusion, we found that a prophylactic dose of propofol prevented EA and decreased the severity of EA in children under sevoflurane anaesthesia. In particular, 3 mg/kg propofol probably provided more pronounced effects without extending the time spent in the PACU, but which requires further RCTs to determine. Furthermore, when patients received an LMA or showed preserved spontaneous breathing, propofol played a more important role in the prevention of EA. Premedication, caudal block, pain, and type of procedure or examination did not influence the process by which propofol prevented EA.

## Data Availability

The raw data supporting the conclusions of this article will be made available by the authors, without undue reservation.
