# Supplementary material for: Efficacy of propofol for the prevention of emergence agitation after sevoflurane anaesthesia in children: A meta-analysis
Source: Front Surg. 2022 Oct 3;9:1031010. doi: 10.3389/fsurg.2022.1031010 (PMC9574203; doi:10.3389/fsurg.2022.1031010)
Supplement: Supplementary file 1 [file Table1.docx]

| Pediatric Anesthesia Emergence Delirium (PAED) scale |
| --- |

|  | Not at all | Just a little | Quite a bit | Very much | Extremely |
| --- | --- | --- | --- | --- | --- |

| Make eye contact with caregiver | 4 | 3 | 2 | 1 | 0 |
| --- | --- | --- | --- | --- | --- |

| Actions are purposeful | 4 | 3 | 2 | 1 | 0 |
| --- | --- | --- | --- | --- | --- |

| Aware of surrounding | 4 | 3 | 2 | 1 | 0 |
| --- | --- | --- | --- | --- | --- |

| Restless | 0 | 1 | 2 | 3 | 4 |
| --- | --- | --- | --- | --- | --- |

| Inconsolable | 0 | 1 | 2 | 3 | 4 |
| --- | --- | --- | --- | --- | --- |
