# Supplementary material for: Efficacy of propofol for the prevention of emergence agitation after sevoflurane anaesthesia in children: A meta-analysis
Source: Front Surg. 2022 Oct 3;9:1031010. doi: 10.3389/fsurg.2022.1031010 (PMC9574203; doi:10.3389/fsurg.2022.1031010)
Supplement: Supplementary file 2 [file Table2.docx]

| **Propofol compared to Control for Emergence agitation in Children accepting sevoflurane anaesthesia** | | | | | | |
| --- | --- | --- | --- | --- | --- | --- |
| **Patient or population:** patients with Emergence agitation in Children accepting sevoflurane anaesthesia **Settings:**  **Intervention:** Propofol **Comparison:** Control | | | | | | |
| **Outcomes** | **Illustrative comparative risks* (95% CI)** | | **Relative effect (95% CI)** | **No of Participants (studies)** | **Quality of the evidence (GRADE)** | **Comments** |
|  | Assumed risk | Corresponding risk |  |  |  |  |
|  | **Control** | **Propofol** |  |  |  |  |
| **Incidence of EA** proportion of children with EA | **Study population** | | **RR 0.51**  (0.38 to 0.67) | 1103 (12 studies) | ⊕⊕⊕⊝ **high**^1,2^ |  |
|  | **524 per 1000** | **267 per 1000** (199 to 351) |  |  |  |  |
|  | **Moderate** | |  |  |  |  |
|  | **534 per 1000** | **272 per 1000** (203 to 358) |  |  |  |  |
| *The basis for the **assumed risk** (e.g. the median control group risk across studies) is provided in footnotes. The **corresponding risk** (and its 95% confidence interval) is based on the assumed risk in the comparison group and the **relative effect** of the intervention (and its 95% CI).  **CI:** Confidence interval; **RR:** Risk ratio; | | | | | | |
| GRADE Working Group grades of evidence **High quality:** Further research is very unlikely to change our confidence in the estimate of effect.  **Moderate quality:** Further research is likely to have an important impact on our confidence in the estimate of effect and may change the estimate. **Low quality:** Further research is very likely to have an important impact on our confidence in the estimate of effect and is likely to change the estimate. **Very low quality:** We are very uncertain about the estimate. | | | | | | |
| ^1^ Moderate heterogeneity but we found causes ^2^ According to the asymmetric funnel plot, there is a publication bias. What's more, all the included studies reported positive results, but by performing trim-and-fill analysis without drawing a diagram, we demonstrated that the results were robust . | | | | | | |
