# Supplementary material for: Efficacy of propofol for the prevention of emergence agitation after sevoflurane anaesthesia in children: A meta-analysis
Source: Front Surg. 2022 Oct 3;9:1031010. doi: 10.3389/fsurg.2022.1031010 (PMC9574203; doi:10.3389/fsurg.2022.1031010)
Supplement: Supplementary file 3 [file Table3.docx]

| **Propofol compared to Control for Emergence agitation in Children accepting sevoflurane anaesthesia** | | | | | | |
| --- | --- | --- | --- | --- | --- | --- |
| **Patient or population:** patients with Emergence agitation in Children accepting sevoflurane anaesthesia **Settings:**  **Intervention:** Propofol **Comparison:** Control | | | | | | |
| **Outcomes** | **Illustrative comparative risks* (95% CI)** | | **Relative effect (95% CI)** | **No of Participants (studies)** | **Quality of the evidence (GRADE)** | **Comments** |
|  | Assumed risk | Corresponding risk |  |  |  |  |
|  | **Control** | **Propofol** |  |  |  |  |
| **PAED scores** PAED scale |  | The mean paed scores in the intervention groups was **3.14 lower** (4.37 to 1.92 lower) |  | 746 (7 studies) | ⊕⊕⊝⊝ **moderate**^1^ |  |
| *The basis for the **assumed risk** (e.g. the median control group risk across studies) is provided in footnotes. The **corresponding risk** (and its 95% confidence interval) is based on the assumed risk in the comparison group and the **relative effect** of the intervention (and its 95% CI).  **CI:** Confidence interval; | | | | | | |
| GRADE Working Group grades of evidence **High quality:** Further research is very unlikely to change our confidence in the estimate of effect.  **Moderate quality:** Further research is likely to have an important impact on our confidence in the estimate of effect and may change the estimate. **Low quality:** Further research is very likely to have an important impact on our confidence in the estimate of effect and is likely to change the estimate. **Very low quality:** We are very uncertain about the estimate. | | | | | | |
| ^1^ Moderate heterogeneity | | | | | | |
