# Supplementary material for: Efficacy of propofol for the prevention of emergence agitation after sevoflurane anaesthesia in children: A meta-analysis
Source: Front Surg. 2022 Oct 3;9:1031010. doi: 10.3389/fsurg.2022.1031010 (PMC9574203; doi:10.3389/fsurg.2022.1031010)
Supplement: Supplementary file 6 [file DataSheet3.docx]

**Appendix 1: Search strategies** (From inception to September 22, 2021)

| No. | PubMed | Results |
| --- | --- | --- |
| #1 | "propofol"[MeSH Terms] | 15943 |
| #2 | "diprivan"[Title/Abstract] OR "fresofol"[Title/Abstract] OR "pofol"[Title/Abstract] OR "propofol"[Title/Abstract] OR "recofol"[Title/Abstract] | 22509 |
| #3 | #1 OR #2 | 24323 |
| #4 | "sevoflurane"[MeSH Terms] | 6729 |
| #5 | "sevofluran*"[Title/Abstract] | 9732 |
| #6 | #4 OR #5 | 10403 |
| #7 | "psychomotor agitation"[MeSH Terms] OR "postoperative complications"[MeSH Terms] OR "confusion"[MeSH Terms] | 609185 |
| #8 | "emergence"[Title/Abstract] AND ("agitation"[Title/Abstract] OR "excit*"[Title/Abstract] OR "delirium"[Title/Abstract] OR "confusion"[Title/Abstract]) | 3446 |
| #9 | ("postoperative"[Title/Abstract] OR "postanaesthetic"[Title/Abstract]) AND ("agitation"[Title/Abstract] OR "confusion"[Title/Abstract] OR "behavioural change*"[Title/Abstract]) | 2183 |
| #10 | #7 OR #8 OR #9 | 612781 |
| #11 | "pediatrics"[MeSH Terms] OR "infant"[MeSH Terms] OR "child"[MeSH Terms] OR "adolescent"[MeSH Terms] | 3839999 |
| #12 | "paediatr*"[Title/Abstract] OR "pediatr*"[Title/Abstract] OR "neonate*"[Title/Abstract] OR "baby"[Title/Abstract] OR "infant*"[Title/Abstract] OR "child*"[Title/Abstract] OR "teenage*"[Title/Abstract] OR "adolescen*"[Title/Abstract] OR "babies"[Title/Abstract] | 2313644 |
| #13 | #11 OR #12 | 4414840 |
| #14 | ("randomized controlled trial"[Publication Type] OR "controlled clinical trial"[Publication Type] OR "randomized"[Title/Abstract] OR "placebo"[Title/Abstract] OR "clinical trials as topic"[MeSH Terms:noexp] OR "randomly"[Title/Abstract] OR "trial"[Title]) NOT ("animals"[MeSH Terms] NOT ("humans"[MeSH Terms] AND "animals"[MeSH Terms])) | 1331071 |
| #15 | #3 AND #6 AND #10 AND #13 AND #14 | 145 |

| No. | Embase | Results |
| --- | --- | --- |
| #1 | 'propofol'/exp OR propofol:ab,ti OR diprivan:ab,ti OR fresofol:ab,ti OR pofol:ab,ti OR recofol:ab,ti | 64331 |
| #2 | 'sevoflurane'/exp OR sevofluran*:ab,ti | 24354 |
| #3 | 'pediatrics'/exp OR 'infant'/exp OR 'child'/exp OR 'adolescent'/exp OR paediatr*:ab,ti OR pediatr*:ab,ti OR neonate*:ab,ti OR bab*:ab,ti OR infant:ab,ti OR child*:ab,ti OR adolescen*:ab,ti OR teenage*:ab,ti | 4820938 |
| #4 | 'restlessness'/exp OR 'postoperative complication'/exp OR 'confusion'/exp OR (emergence:ab,ti AND (agitation:ab,ti OR excit*:ab,ti OR delirium:ab,ti OR confusion:ab,ti)) OR ((postoperative:ab,ti OR postanaesthetic:ab,ti) AND (agitation:ab,ti OR confusion:ab,ti OR 'behavior change*':ab,ti)) | 847985 |
| #5 | 'crossover procedure':de OR 'double-blind procedure':de OR 'randomized controlled trial':de OR 'single-blind procedure':de OR random*:de,ab,ti OR factorial*:de,ab,ti OR crossover*:de,ab,ti OR ((cross NEXT/1 over*):de,ab,ti) OR placebo*:de,ab,ti OR ((doubl* NEAR/1 blind*):de,ab,ti) OR ((singl* NEAR/1 blind*):de,ab,ti) OR assign*:de,ab,ti OR allocat*:de,ab,ti OR volunteer*:de,ab,ti | 2902242 |
| #6 | #1 AND #2 AND #3 AND #4 AND #5 | 314 |

| No. | Cochrane Central Register of Controlled Trials | Results |
| --- | --- | --- |
| #1 | MeSH descriptor: [Propofol] explode all trees | 5150 |
| #2 | (propofol):ti,ab,kw OR (diprivan):ti,ab,kw OR (fresofol):ti,ab,kw OR (pofol):ti,ab,kw OR (recofol):ti,ab,kw | 15093 |
| #3 | #1 OR #2 | 15093 |
| #4 | MeSH descriptor: [Sevoflurane] explode all trees | 2267 |
| #5 | (sevofluran*):ti,ab,kw | 6195 |
| #6 | #4 OR #5 | 6195 |
| #7 | MeSH descriptor: [Pediatrics] explode all trees | 721 |
| #8 | MeSH descriptor: [Infant] explode all trees | 34437 |
| #9 | MeSH descriptor: [Child] explode all trees | 60484 |
| #10 | MeSH descriptor: [Adolescent] explode all trees | 109543 |
| #11 | (paediatr*):ti,ab,kw OR (pediatr*):ti,ab,kw OR (neonate*):ti,ab,kw OR (baby):ti,ab,kw OR (babies):ti,ab,kw | 54979 |
| #12 | (infant*):ti,ab,kw OR (child*):ti,ab,kw OR (teenage*):ti,ab,kw OR (adolescen*):ti,ab,kw | 300272 |
| #13 | #7 OR #8 OR #9 OR #10 OR #11 OR #12 | 311383 |
| #14 | MeSH descriptor: [Psychomotor Agitation] explode all trees | 1086 |
| #15 | MeSH descriptor: [Postoperative Complications] explode all trees | 42919 |
| #16 | MeSH descriptor: [Confusion] explode all trees | 1073 |
| #17 | (emergence):ti,ab,kw | 5507 |
| #18 | (agitation):ti,ab,kw OR (excit*):ti,ab,kw OR (delirium):ti,ab,kw OR (confusion):ti,ab,kw | 17197 |
| #19 | #17 AND #18 | 1237 |
| #20 | (postoperative):ti,ab,kw OR (postanaesthetic):ti,ab,kw | 130036 |
| #21 | (agitation):ti,ab,kw OR (confusion):ti,ab,kw OR (behavioural change*):ti,ab,kw | 28010 |
| #22 | #20 AND #21 | 1787 |
| #23 | #14 OR #15 OR #16 OR #19 OR #22 | 46193 |
| #24 | #3 AND #6 AND #13 AND #23 | 268 |
| #25 | Trials matching #24 | 265 |

| No. | ClinicalTrials.gov | Results |
| --- | --- | --- |
| #1 | propofol \| Agitation, Emergence \| Child | 20 |
