# Supplementary figures and images for: Efficacy of propofol for the prevention of emergence agitation after sevoflurane anaesthesia in children: A meta-analysis
Source: Front Surg. 2022 Oct 3;9:1031010. doi: 10.3389/fsurg.2022.1031010 (PMC9574203; doi:10.3389/fsurg.2022.1031010)

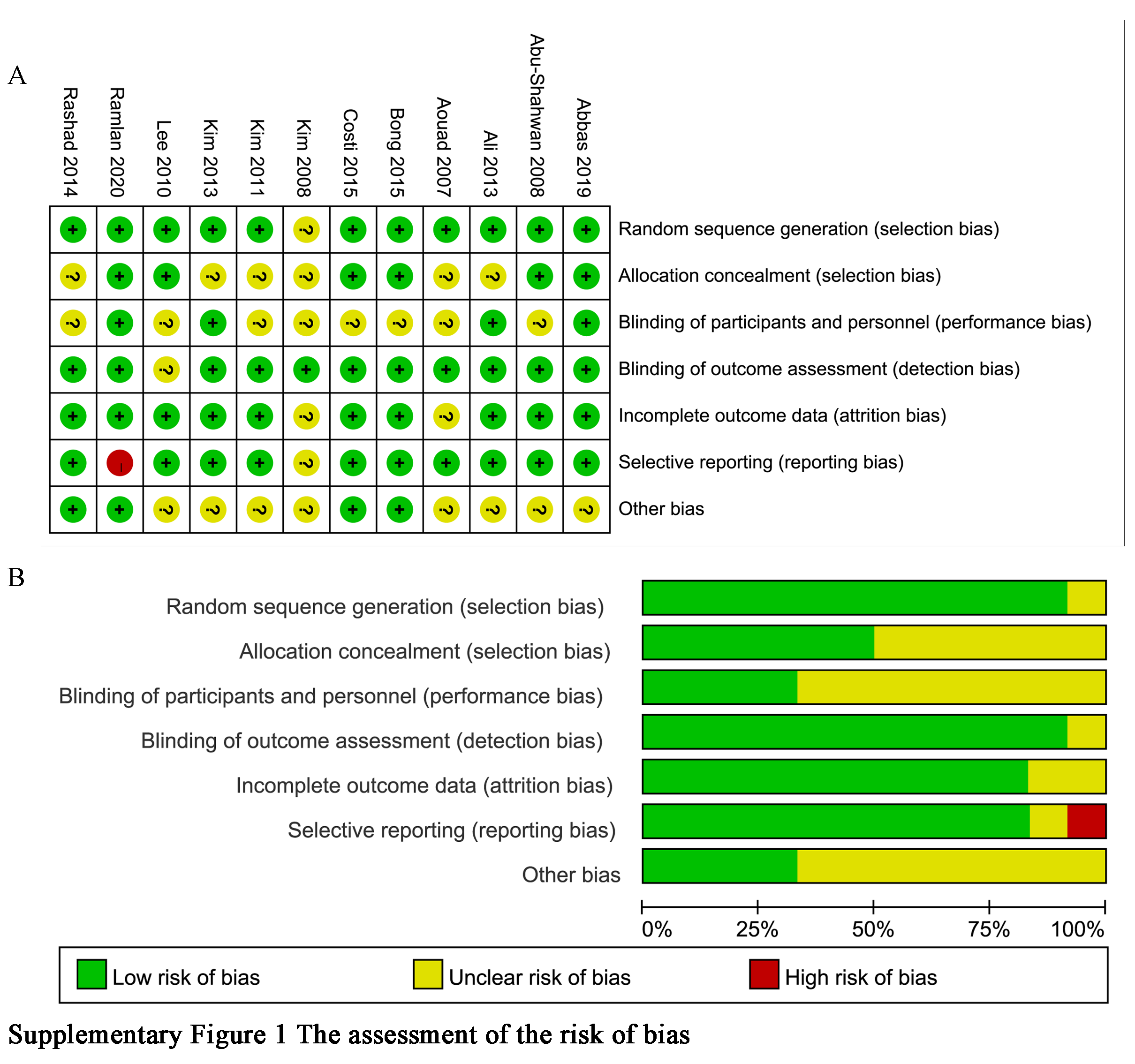

Supplement: Supplementary file 7 [file Image1.tif]

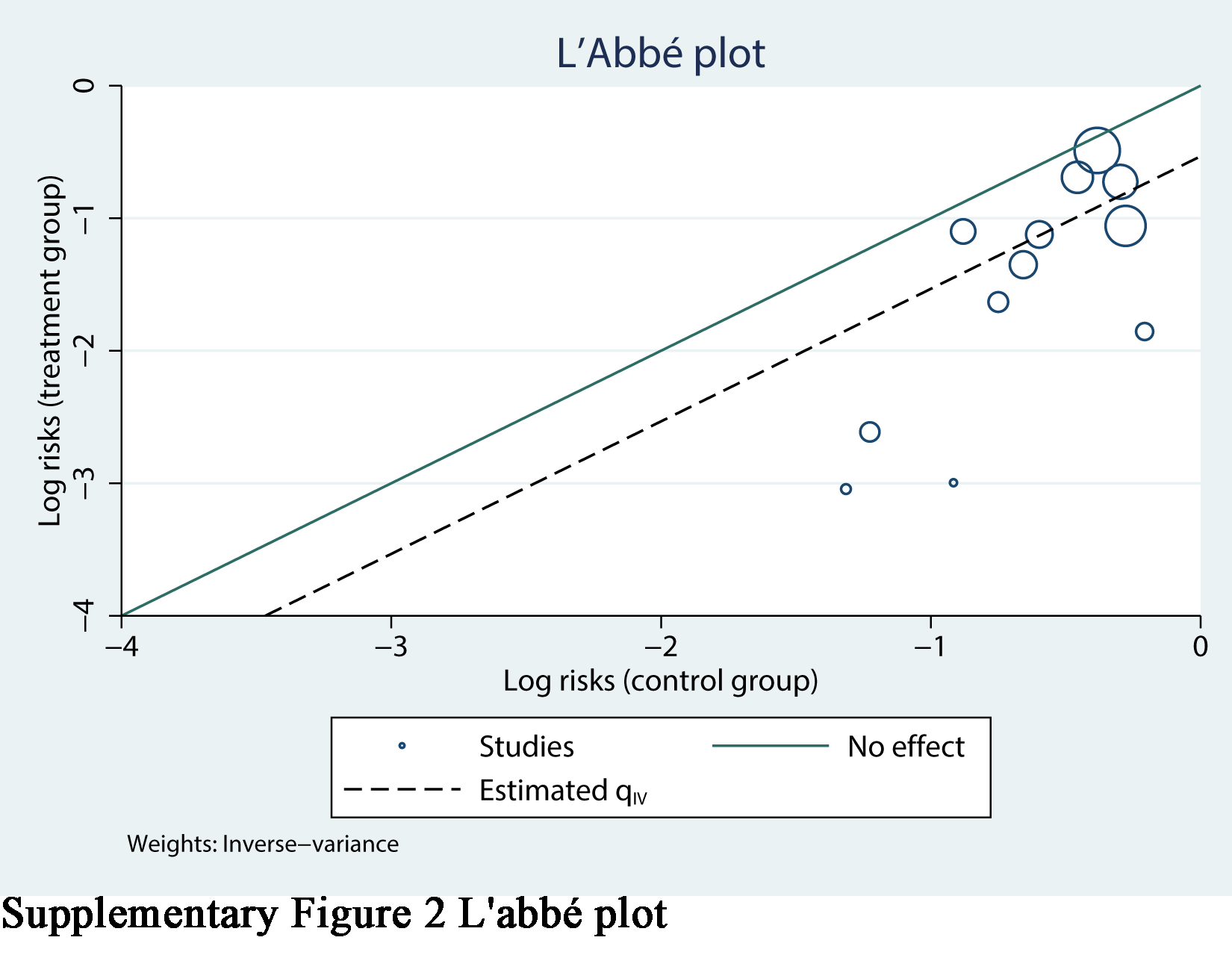

Supplement: Supplementary file 8 [file Image2.tif]

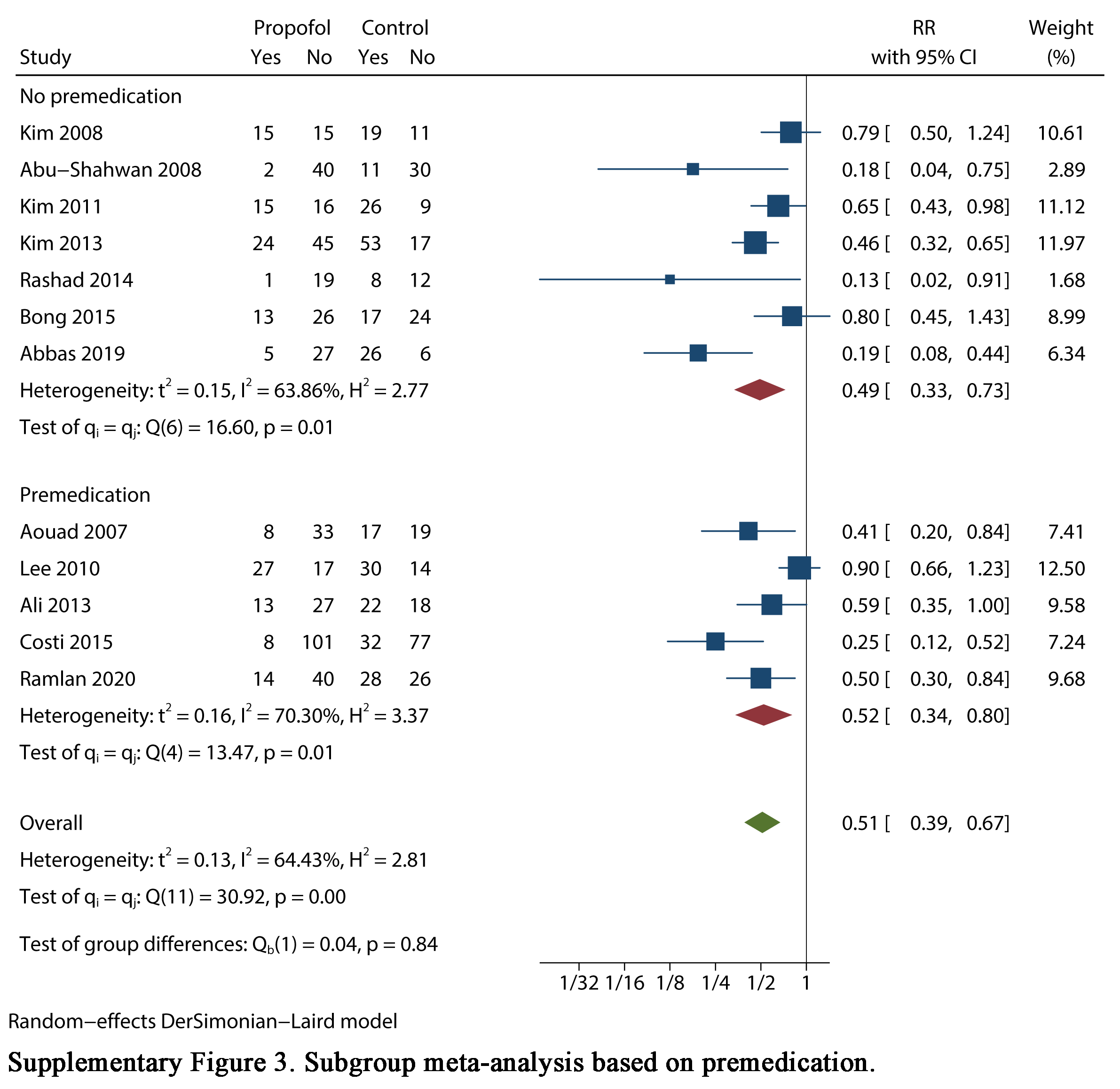

Supplement: Supplementary file 9 [file Image3.tif]

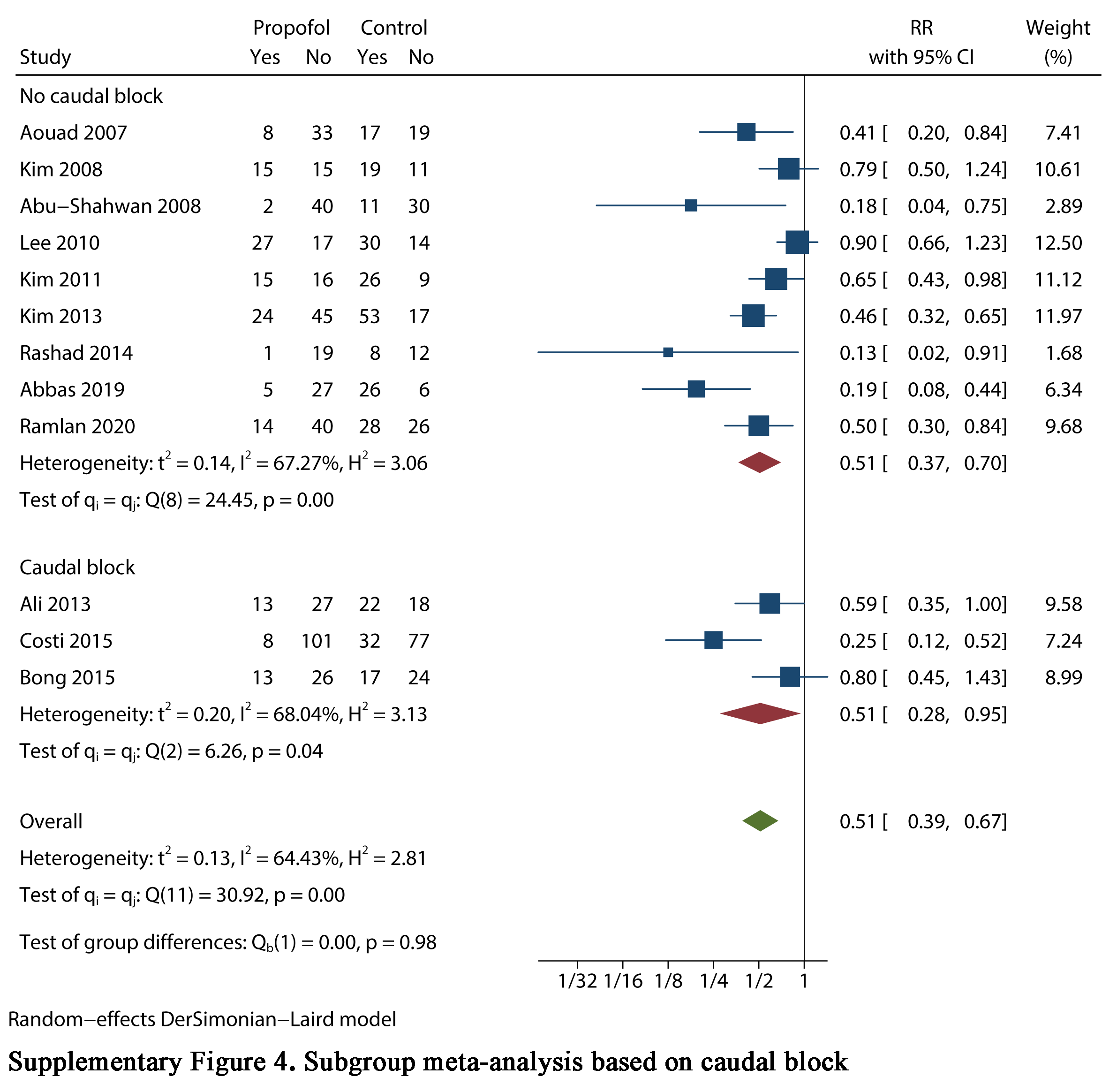

Supplement: Supplementary file 10 [file Image4.tif]

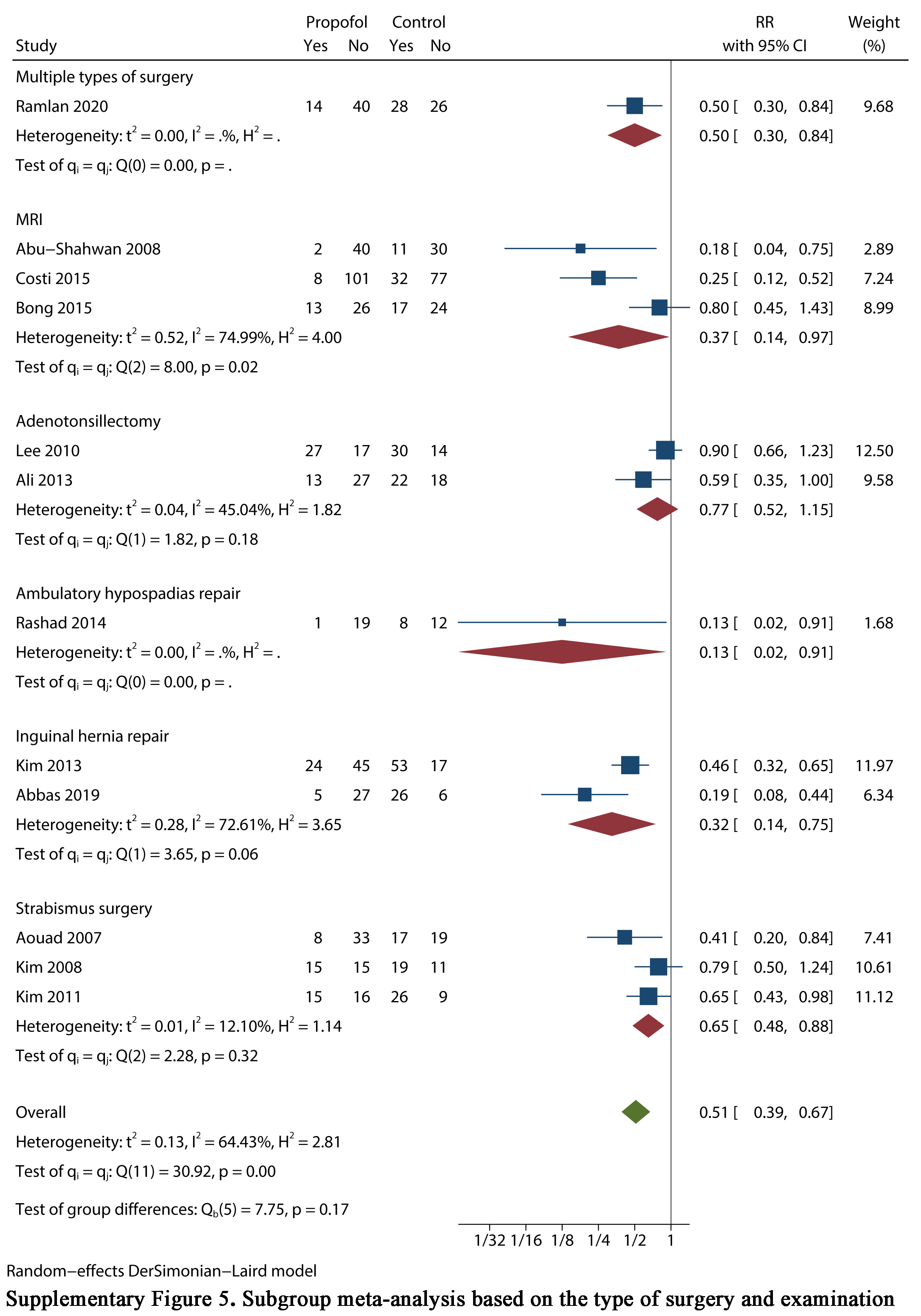

Supplement: Supplementary file 11 [file Image5.tif]

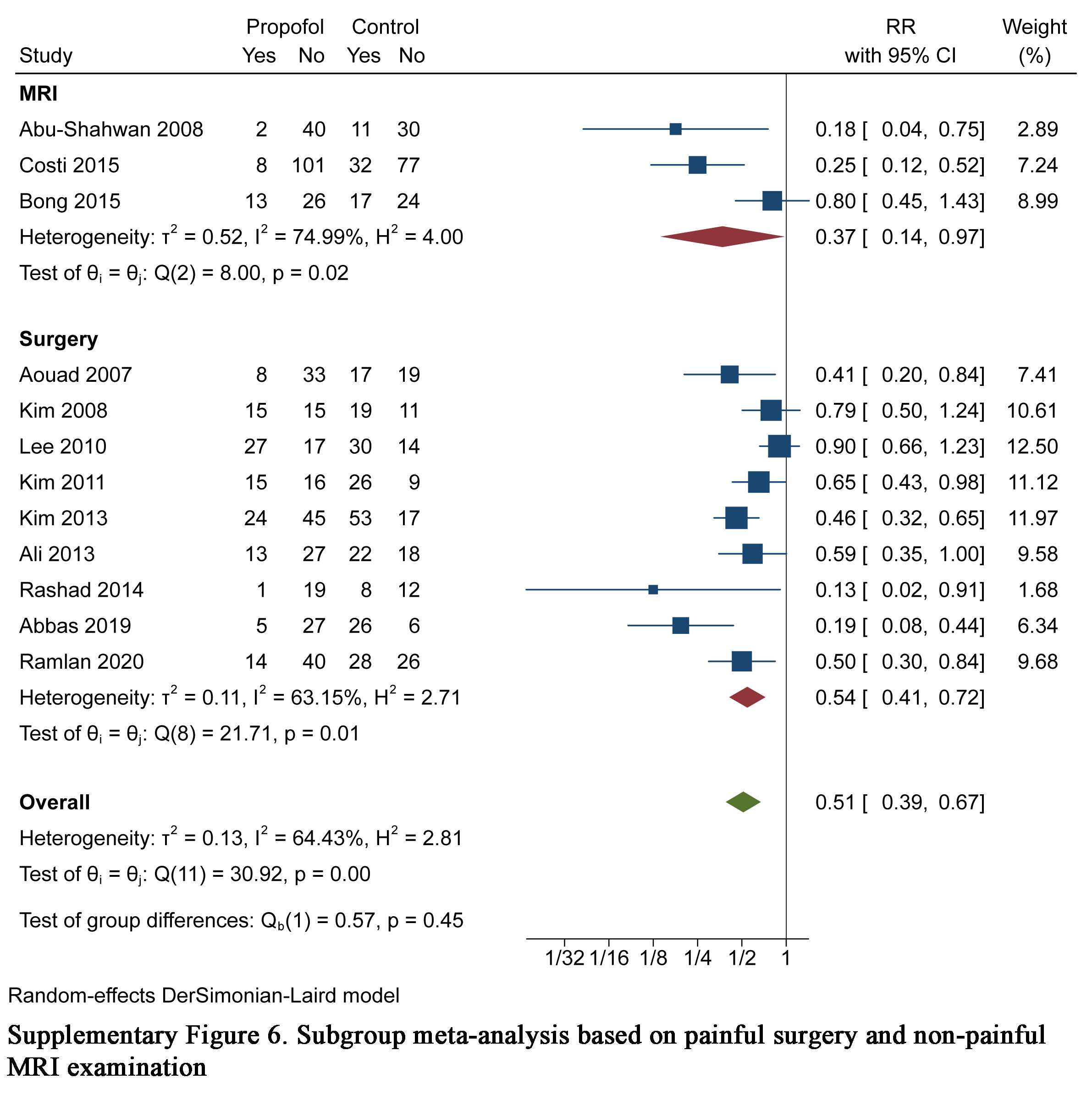

Supplement: Supplementary file 12 [file Image6.tif]
